# Self-Powered Strain Sensing System: A Cutting-Edge Review Paving the Way for Autonomous Wearable Electronics

**DOI:** 10.3390/polym17243256

**Published:** 2025-12-06

**Authors:** Hui Song

**Affiliations:** School of Materials Science and Engineering, Linyi University, Linyi 276000, China; songhui@lyu.edu.cn

**Keywords:** wearable electronics, self-powered systems, strain sensors, flexible energy storage devices, nanogenerators

## Abstract

Self-powered strain sensing technology represents a pivotal frontier in overcoming the energy constraints of wearable electronics, thereby enabling their long-term intelligence and operational autonomy. This review systematically summarizes recent advances in integrated strain sensing systems, with a particular focus on three primary strategies for achieving self-powered functionality: integration with energy storage devices (e.g., flexible supercapacitors and microbatteries); integration with energy harvesters (e.g., triboelectric and piezoelectric nanogenerators); and advanced systems that synergistically combine energy harvesting, storage, and management modules. The article begins by outlining the fundamental working mechanisms and key performance parameters of strain sensors. It then provides a detailed analysis of the material systems, innovative structural designs, operational mechanisms, and applications in health monitoring and human-computer interaction associated with the different self-powered strategies. Finally, the review critically examines the persistent challenges in this field, including energy balance, mechanical robustness, and environmental stability, and offers perspectives on future research directions such as multimodal energy harvesting, intelligent data processing, and the development of biocompatible materials. This work aims to serve as a valuable reference for advancing the practical implementation of truly autonomous and wearable strain sensing systems.

## 1. Introduction

**Figure 1:**
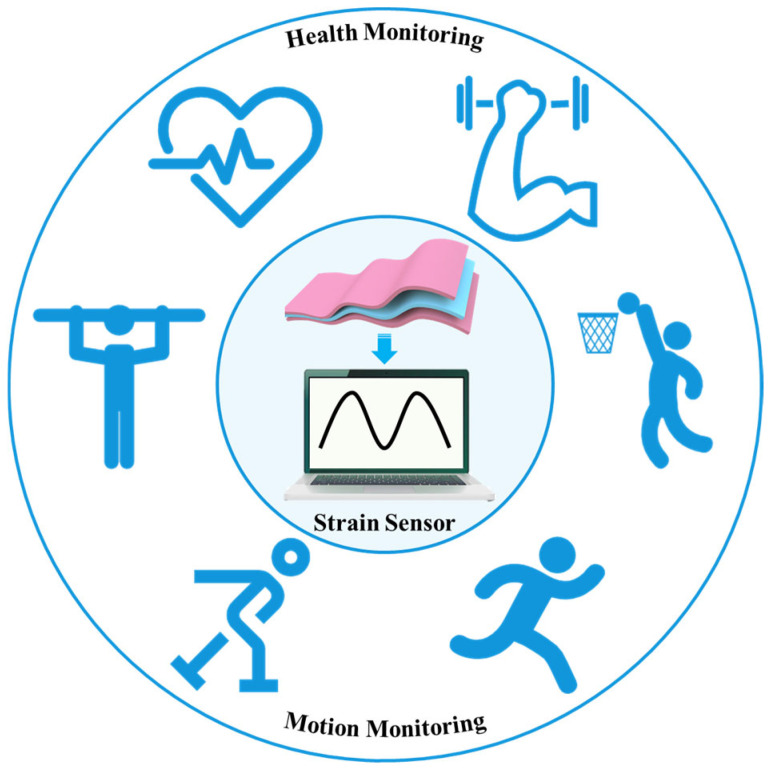
The application of strain sensors in human motion monitoring and health monitoring.

In recent years, the rapid advancement of the Internet of Things (IoT), artificial intelligence (AI), and big data technologies has accelerated the transition of wearable electronic devices from laboratory concepts to commercialization and real-world applications. Given their exceptional portability, multifunctionality, and intelligence, such devices have been widely adopted in areas including personal health monitoring, sports science analysis, and flexible human–computer interaction interfaces [1]. A core function of these systems is the continuous, stable, and unobtrusive detection of physiological or environmental parameters, effectively converting them into processable and analyzable digital signals. This capability provides essential data for health assessment, activity recognition, and ecological awareness [2]. Among various sensing mechanisms, strain sensors have garnered significant attention due to their ability to directly and sensitively detect mechanical deformations. Their suitability for capturing mechanical signals generated by human motion and physiological activities (Figure 1). For instance, critical physiological activities—such as the bending of joints, stretching of muscle tissue, and periodic pulsation of blood vessels—can induce structural deformation in the sensor, resulting in predictable variations in its electrical properties, such as resistance or capacitance [3,4]. By extracting and analyzing these signal variations, the system can non-invasively acquire important user information, including movement status, muscle load, and heart rate [5]. Therefore, strain sensors not only play a key role in motion posture recognition and rehabilitation training but also serve as a core sensing component for constructing comfortable, conformal, and high signal-to-noise ratio flexible bioelectronic systems. Consequently, they represent fundamental hardware for enabling wearable technology to achieve accurate perception and intelligent feedback.

An ideal strain sensor should exhibit not only high sensitivity, a wide sensing range, fast response/recovery times, and excellent cyclic stability but also favorable flexibility, stretchability, breathability, and conformal adhesion to human skin to meet the demands of practical wearable applications [6,7]. In recent years, remarkable progress has been made in enhancing the overall performance of strain sensors through innovative micro/nano structures—such as wrinkled geometries [8] and sponge-like porous architectures [9]—as well as the adoption of advanced functional materials like carbon nanomaterials [6], metal nanowires [10,11], and conductive polymers [12]. However, in practice, most high-performance strain sensors are active devices that require a continuous external power supply for signal conversion and transmission [13,14]. Conventional rigid batteries, due to their bulkiness, limited flexibility, and finite lifespan, are inadequate for next-generation wearable electronics that demand lightweight, flexible, and long-term stable power [15]. Frequent recharging or battery replacement not only compromises user experience but may also lead to the loss of critical physiological data during power interruptions. Thus, developing sustainable and maintenance-free energy solutions is a crucial challenge for achieving genuine autonomy and intelligence in wearable electronics.

In this context, self-powered technology has emerged as a promising pathway to address the energy constraints of wearable devices, becoming a key research focus. The core concept of self-powered sensing systems involves harvesting energy from the human body or the ambient environment and converting it into usable electricity for powering the sensors, thereby enabling continuous operation without external power sources [16,17]. Currently, technical approaches for realizing self-powered strain sensing systems can be broadly categorized into three types: (1) Integration with energy storage systems, which involve combining flexible supercapacitors, micro-batteries, or other micro-energy-storage units with strain sensors [18,19]. Such systems deliver stable energy output, though the storage units themselves still require periodic charging from the external environment. (2) Integration with energy harvesters, where nanogenerators or other energy harvesting devices are combined with strain sensors, allowing the sensor to generate electrical output or be directly powered during deformation [20,21]. This approach offers the advantage of real-time energy harvesting and utilization, though its output is often pulsed and relatively unstable. (3) Energy-managed integrated systems, which incorporate energy harvesters, storage units, and sensors into a unified microsystem through rational structural design and power management circuits [22]. This architecture enables efficient energy harvesting, storage, and distribution, representing the most promising solution for achieving long-term, stable, and truly self-powered operation.

**Figure 2:**
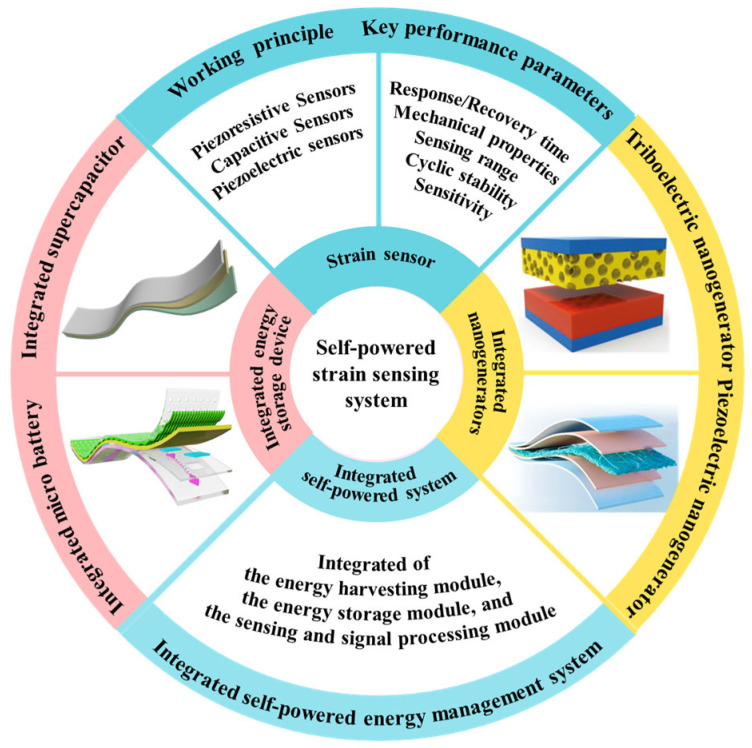
Summary overview chart. (Reproduced from [24,25,26] with permission from Royal Society of Chemistry, Wiley, and Elsevier).

This review systematically examines recent research advances in three self-powered strain sensing approaches: integrated energy storage devices, integrated energy harvesters, and integrated energy management systems (Figure 2). Following an overview of the fundamental working principles and key performance parameters of strain sensors, separate sections elaborate on the operational mechanisms, material systems, innovative structural designs, and representative wearable applications—such as health monitoring and human-computer interaction—associated with each approach. Finally, this review critically examines the significant challenges facing current technology and outlines future research directions, seeking to provide a systematic reference for researchers working in the field of self-powered flexible sensing. Compared to existing reviews on strain sensors and self-powered systems [23], this review offers a focused analysis by systematically categorizing and comparing three primary integration strategies—energy storage, energy harvesting, and energy-managed systems—with emphasis on system-level design, energy autonomy, and real-world applicability. We also present a comprehensive performance comparison of recent self-powered strain sensors (Table 1) and discuss emerging trends such as intelligent energy management, multimodal energy harvesting, and biodegradable electronics, which are pivotal for advancing the next generation of autonomous wearable systems.

## 2. Strain Sensors

Prior to discussing self-powered systems, it is essential to systematically introduce the fundamental concepts and key characteristics of strain sensors. A strain sensor is fundamentally a transducer that converts mechanical deformation (i.e., strain, ε) into measurable electrical signals such as resistance (R), capacitance (C), or charge (Q). Its performance is a critical determinant of the accuracy and reliability of the overall sensing system.

### 2.1. Working Mechanisms

Based on differences in signal transduction principles, strain sensors can be primarily classified into three types: piezoresistive [27], capacitive [28], and piezoelectric [29]. Each type exhibits distinct characteristics in terms of working mechanism, performance, and suitable application scenarios.

#### 2.1.1. Piezoresistive Sensors

**Figure 3:**
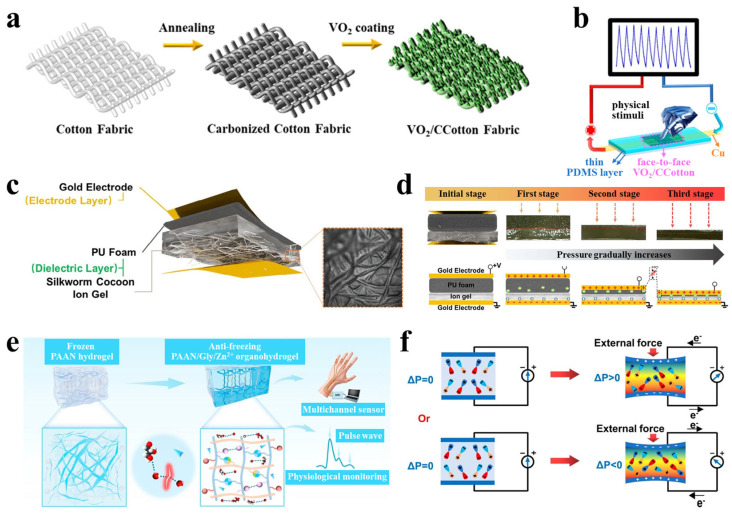
(**a**) Fabrication procedure of VO_2_/CCotton. (**b**) The VO_2_/CCotton-based flexible piezoresistive sensor. (Reproduced from [33] with permission from ACS Publications). (**c**) Schematic of the pressure sensor with a dual-dielectric-layer design. (**d**) Micromorphology and sensing mechanism of the pressure sensor under varying pressures. (Reproduced from [37] with permission from Wiley). (**e**) Illustration and application scenarios of the antifreezing self-powered organohydrogel. (**f**) Working mechanism of stress-induced polarization generating electrical signals in an external circuit. (Reproduced from [44] with permission from ACS Publications).

As the most widely used type, piezoresistive sensors operate based on the change in electrical resistance of conductive materials or structures under mechanical strain [30]. When the sensor is stretched or compressed, its internal conductive pathways undergo geometric deformation, changes in contact points, or crack propagation, leading to an increase in resistance. Upon release of the strain, the conductive network recovers, and the resistance decreases accordingly [31,32]. For instance, Han et al. [33] successfully fabricated a dual-functional textile (VO_2_/CCotton) with integrated piezoresistive sensing and energy storage capabilities. This was achieved by growing vanadium dioxide nanosheets in situ on a carbonized cotton fabric substrate via a simple hydrothermal strategy (Figure 3a). From a sensing mechanism perspective, this sensor operates on the typical piezoresistive effect. When external pressure is applied to the two-layer VO_2_/CCotton structure, the three-dimensional conductive network formed by VO_2_ nanosheets and conductive carbon fibers deforms. This significantly increases the number and contact area among conductive pathways, resulting in reduced resistance and increased current (Figure 3b). The unique metal–insulator phase transition characteristic of VO_2_ nanosheets further enhances the electrical response sensitivity. Leveraging this mechanism, the sensor demonstrates high sensitivity (up to 7.12 kPa^−1^ in the 0–2.0 kPa range), a broad sensing range (0–120 kPa), excellent response speed (response/recovery times of 12 ms and 8 ms, respectively), and stable performance over more than 5000 cycles. This study not only validates its reliability in monitoring human physiological signals and joint movements but also highlights the integration of a piezoresistive sensing unit with a flexible energy storage device, offering a promising material and structural design strategy for self-powered wearable systems. Despite advantages such as simple signal readout, high sensitivity, and the ability to detect minor strains, most piezoresistive sensors require a continuous external voltage to monitor resistance changes, leading to relatively high static power consumption. They are also prone to sensitivity degradation and nonlinear responses under large or cyclic strains. To mitigate the high static power consumption associated with conventional piezoresistive sensors, emerging strategies focus on passive or quasi-passive operation modes. For instance, Zhang et al. [34] recently proposed a bioinspired potentiometric strain sensor that eliminates the need for continuous external excitation. This sensor utilizes an ionic percolating network within a stretchable composite electrolyte, coupled with a pair of electrochemically active electrodes (Al and carbon), to generate a strain-modulated potential difference. Since no external voltage is required for signal generation, the device operates with ultralow static power consumption at the nanowatt level while maintaining the capability to detect both static and dynamic strains. This approach exemplifies a promising direction for developing energy-efficient piezoresistive-like sensors by shifting from traditional resistive readout to self-generated potentiometric signaling.

#### 2.1.2. Capacitive Sensors

Capacitive sensors typically adopt a parallel-plate capacitor structure, where the capacitance is determined by the dielectric layer between the electrodes and the geometric dimensions. The fundamental relationship is expressed as *C = ε*_0_*ε_r_A/d*, where *ε*_0_ is the vacuum permittivity, *ε_r_* is the relative permittivity of the dielectric, *A* is the overlapping electrode area, and *d* is the distance between the electrodes. Mechanical strain induces capacitance variations by altering either *A* or *d* [35,36]. Pu et al. [37] developed an ionic pressure sensor based on a dual-dielectric layer structure. The sensing mechanism of this device combines the parallel-plate capacitance principle with an interfacial electric double-layer capacitance effect. The sensor employs silk cocoon-derived ionogel as a high-permittivity layer and open-cell polyurethane foam as a low-permittivity layer, forming a dual-dielectric configuration (Figure 3c). Under applied pressure, the polyurethane foam compresses first, reducing the inter-electrode distance. As pressure increases, free ions from the ionogel penetrate the foam and contact the gold electrode, forming an effective electric double layer that substantially increases the interfacial contact area (Figure 3d). The synergistic effect of reduced distance d and significantly enhanced effective area A amplifies the capacitance response by several orders of magnitude, enabling ultra-high sensitivity (72,548.7 kPa^−1^) and a broad detection range (0.001–420 kPa). This work, therefore, establishes a pioneering iontronic sensing paradigm that merges geometric capacitance with interfacial ionic dynamics. This dual-dielectric and ionic migration strategy transcends conventional capacitive design, underpinning the device’s multimodal and ultra-sensitive capabilities. Capacitive sensors offer advantages such as low power consumption, temperature insensitivity, and suitability for static strain measurement. However, they are susceptible to environmental electromagnetic interference, generally exhibit lower sensitivity compared to piezoresistive sensors, and may suffer from measurement inaccuracies due to interfacial gap variations when attached to human skin.

#### 2.1.3. Piezoelectric Sensors

Piezoelectric sensors utilize the piezoelectric effect exhibited by materials such as polyvinylidene fluoride (PVDF) [38,39] and zinc oxide (ZnO) [40,41], which generate electrical charges in response to applied stress. These sensors can directly convert dynamic strain into electrical signals without requiring an external power supply, thereby exhibiting inherent “self-powering” potential [42,43]. Shi et al. [44] developed a flexible wearable sensor based on piezoelectric organic hydrogels, whose operating mechanism relies on the polarization of PVDF under mechanical stress and the consequent generation of potential differences, enabling self-powered signal output (Figure 3e,f). Unlike conventional piezoelectric hydrogels, this system significantly promotes the transition of PVDF from the non-polar α-phase to the electrically active β-phase by introducing dipole interactions between PAN chains and PVDF chains within the polymer network. This structural optimization achieved a piezoelectric constant (d_33_) of 35 pC/N. Furthermore, the sensor demonstrates remarkable mechanical properties, including high tensile strength (780%), superior toughness (8.23 MJ·m^−3^), and excellent fatigue resistance, making it suitable for interfacing with human skin. The device has been successfully applied in various scenarios, including human motion monitoring, speech recognition, pulse detection, and Morse code communication. Beyond conventional piezoelectric materials, perovskite oxides have emerged as promising candidates. For instance, Wang et al. recently demonstrated that cation defect engineering—specifically introducing Cu vacancies via thermal-driven resolution in CaCu_3_Ti_4_O_12_—can induce significant piezoelectricity (d_33_ ≈ 7 pC N^−1^) in otherwise centrosymmetric quadruple perovskites. This approach not only creates piezoelectricity but also enables piezo-photocatalytic functionality, highlighting its potential for self-powered sensing systems [45]. While piezoelectric sensors exhibit high sensitivity to high-frequency dynamic signals such as vibrations and impacts, they cannot maintain stable output under continuous static strain due to inherent charge leakage. Additionally, their characteristic alternating-current pulse output generally requires specialized signal conditioning circuits for effective acquisition and processing. To suppress charge leakage and enhance the static output stability of polymer-based PENGs, researchers have pursued optimization through signal compensation strategies. For instance, Roshandel et al. [46] proposed a modified active Prandtl–Ishlinskii model for low-impedance piezoelectric actuators. By incorporating nonlinear functions to characterize the internal resistance, the model effectively estimates and compensates for signal attenuation due to charge leakage. This approach, originally developed for self-sensing positioning in actuators, also offers viable circuit and signal-processing strategies for enabling stable PENG output under static or quasi-static strain monitoring.

#### 2.1.4. Comparison of Sensing Mechanisms

**Figure 4:**
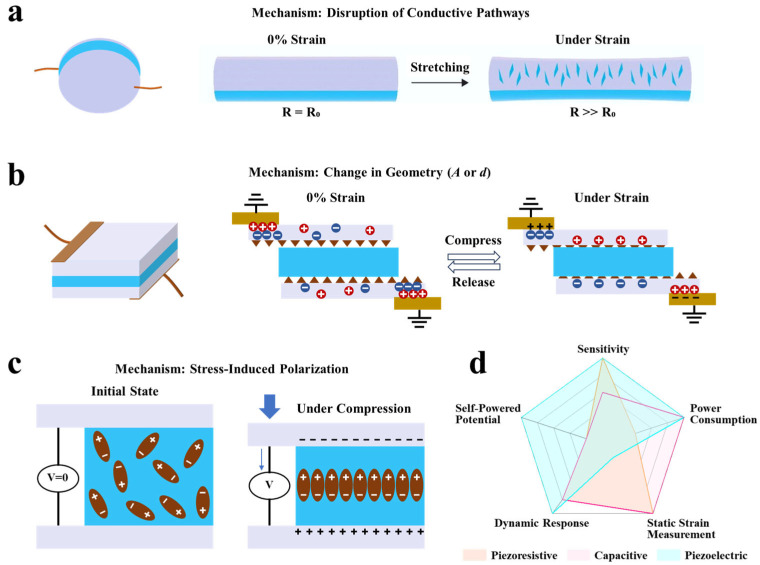
Strain sensing mechanism diagrams of (**a**) piezoresistive, (**b**) capacitive, and (**c**) piezoelectric sensors. (**d**) Performance radar chart of piezoresistive, capacitive, and piezoelectric sensors.

The choice of sensing mechanism is fundamental to the design of a strain sensor, as each principle offers distinct advantages and faces specific limitations, making them uniquely suited for different application scenarios (Figure 4). Piezoresistive sensors, benefiting from straightforward signal readout and high sensitivity—particularly for minor strains—are widely adopted [47]. However, their reliance on an external power source leads to continuous energy consumption, and they often suffer from hysteresis and nonlinear responses under large or cyclic deformation, which can limit long-term stability. Capacitive sensors excel with their low power consumption, minimal hysteresis, and capability for static strain measurement. Their main drawbacks include generally lower sensitivity compared to piezoresistive counterparts, susceptibility to electromagnetic interference, and potential signal inaccuracies caused by variations in the electrode-skin interface distance in wearable configurations [48]. In contrast, piezoelectric sensors possess an inherent self-powering capability, generating electrical signals directly from dynamic mechanical stimuli without an external power supply, and they exhibit high sensitivity and rapid response to high-frequency events like vibrations and impacts [49]. Nevertheless, their inability to measure static strains due to inherent charge leakage, the pulsed nature of their output requiring specialized signal conditioning circuits, and the frequent trade-off between high piezoelectric coefficients and mechanical flexibility (e.g., in ceramic materials) represent significant challenges for broader application.

This comparative analysis underscores that no single sensing mechanism is universally superior. The optimal selection hinges on a careful balance of application-specific requirements, including the nature of the strain (static vs. dynamic), the necessity for self-powering, desired sensitivity, power availability, and the operational environment. A thorough understanding of these fundamental principles thus provides a critical foundation for the subsequent design and integration of self-powered strain sensing systems, as will be discussed in the following sections.

### 2.2. Key Performance Parameters

A comprehensive evaluation of strain sensor performance necessitates the consideration of several key parameters, including sensitivity (gauge factor, *GF*), sensing range, response/recovery time, cyclic stability, and mechanical properties (e.g., elastic modulus and elongation at break).

#### 2.2.1. Sensitivity (GF)

**Figure 5:**
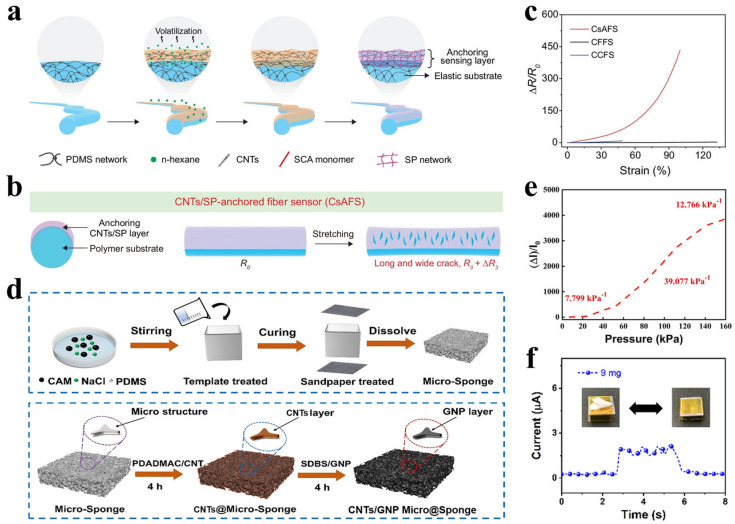
(**a**) Fabrication process of the fiber strain sensor based on the in-situ formation of anchored sensing materials. (**b**) Crack formation mechanism in the proposed CNTs/SP-anchored fiber sensor (CsAFS). (**c**) Relative resistance changes of the CsAFS under tensile strain. (Reproduced from [53] with permission from OXFORD Academic). (**d**) Schematic of the CNTs/GNP Micro@Sponge fabrication procedure. (**e**) Current response curve under pressures ranging from 0 to 160 kPa. (**f**) Real-time response signal of the pressure sensor to a piece of paper (9 mg). (Reproduced from [56] with permission from ScienceDirect).

Sensitivity, typically quantified by the gauge factor (*GF*), is defined as *GF* = (∆*R*/*R*_0_)/*ε*_0_, representing the relative change in electrical resistance per unit strain. A higher *GF* value indicates a greater ability to detect minute deformations [50]. Piezoresistive sensors typically exhibit high *GF* values, which can be further enhanced through structural designs such as microcrack generation or multilevel architectures [51,52]. Illustratively, Yu et al. [53] significantly improved the performance of a fiber strain sensor by in situ construction of an ultra-mechanosensitive anchored sensing layer. This approach utilized the hydrolysis and condensation of silane coupling agents on the fiber surface to form a firmly bonded carbon nanotube/siloxane polymer (CNTs/SP) composite sensing layer (Figure 5a). The significant mechanical mismatch between this layer and the elastic PDMS substrate promotes the formation of longer and wider microcracks during stretching, leading to a sharp resistance change (Figure 5b). Based on this mechanism, the fabricated sensor achieved a *GF* of up to 433.6 within a 100% strain range—approximately 254 times more sensitive than conventional filled-type sensors—along with an ultralow detection limit of 0.05% and excellent cycling durability (Figure 5c). This strategy provides an effective material and interface engineering approach for developing highly sensitive wearable sensors.

#### 2.2.2. Sensing Range

The sensing range refers to the maximum strain over which the sensor operates with predictable and reliable effectiveness. Human activities induce strains of varying magnitudes—for instance, pulse waveforms below 1%, finger bending around 30%, and joint movements exceeding 50%. An ideal sensor should therefore combine high sensitivity with a broad sensing range [54,55]. Ni et al. [56] successfully fabricated a flexible piezoresistive sensor with both high sensitivity and a wide detection range through the strategic combination of sacrificial templating and surface microstructure engineering (Figure 5d). Using NaCl and citric acid monohydrate as co-sacrificial templates, they constructed an elastic PDMS sponge with a hierarchical porous structure and further induced irregular microstructures on its surface via sandpaper templating to increase the initial contact area. Subsequently, a conductive layer was formed by layer-by-layer self-assembly of carbon nanotubes and graphene nanosheets. The resulting sensor exhibited a remarkable sensitivity of 39.077 kPa^−1^ in the medium-pressure regime (50–110 kPa), with a detection range spanning from subtle stimuli as low as 0.9 Pa up to 160 kPa (Figure 5e,f). This broad response capability enables stable monitoring of diverse physiological and motion activities—from weak signals such as pulse and respiration to finger bending and even foot stepping—offering an effective strategy to overcome the typical trade-off between sensitivity and sensing range.

#### 2.2.3. Response/Recovery Time

**Figure 6:**
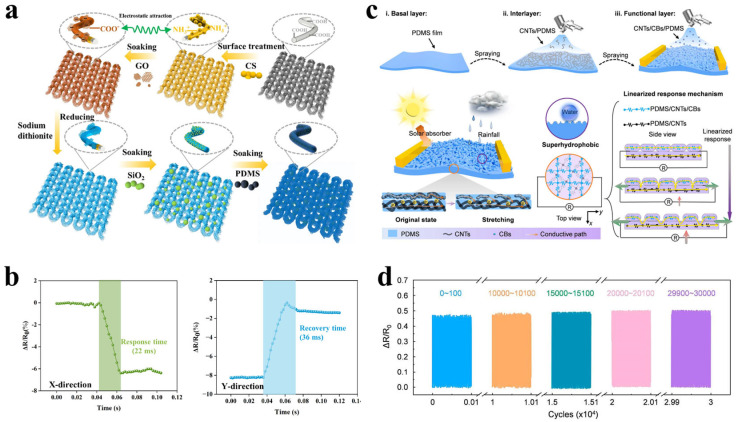
(**a**) Preparation process of the PSKF@rGO/SiO_2_-PDMS sensor. (**b**) Response speed and recovery time of the PSKF@rGO/SiO_2_-PDMS fabric under 5% deformation in the X and Y direction. (Reproduced from [59] with permission from Springer Nature). (**c**) Design, preparation, and mechanism illustration of the bio-inspired bionic sensor. (**d**) Cycling performance of the sensor under 25% tensile strain for 30,000 cycles. (Reproduced from [62] with permission from ScienceDirect).

Response and recovery time refer to the time required for a sensor to transition from its initial state to a stable response state, and to return from the response state to the initial state, respectively. Short response and recovery times are critical for capturing rapid and continuous movements, such as muscle vibrations or speech signals [57,58]. For instance, Lu et al. [59] developed a superhydrophobic fabric strain sensor based on reduced graphene oxide (rGO), which exhibited excellent rapid response characteristics, making it highly suitable for human motion monitoring. In this study, a conductive functional layer composed of rGO/SiO_2_-PDMS (PSKF@rGO/SiO_2_-PDMS) was constructed on polyamide/spandex knitted fabric via electrostatic self-assembly and dip-coating processes (Figure 6a). Test results demonstrated that the sensor achieved a response time of 22 ms and a recovery time of 36 ms under 5% strain (Figure 6b). This remarkable dynamic performance is attributed to the unique coiled structure of the fabric. During stretching, yarn segments slip, causing numerous fibers to compress against each other, thereby rapidly altering the contact resistance and enabling a fast response to external strain. Even after 4000 cycles, the sensor maintained a stable response signal, confirming its reliability for real-time and continuous monitoring of human joint movements and other applications.

#### 2.2.4. Cyclic Stability

Cyclic stability quantifies the ability of a sensor to maintain consistent performance over extended periods under repeated loading and unloading cycles. It is typically assessed by the degree of signal attenuation after hundreds to thousands of cycles under a specific strain [60,61]. Good cyclic stability is essential for practical wearable applications. Inspired by the multifunctional properties of moth wings, Li et al. [62] fabricated a bio-inspired superhydrophobic strain sensor using layer-by-layer spraying and interfacial curing techniques (Figure 6c). The sensor features a dual-conductive layer structure composed of quasi-homogeneous materials (a PDMS/CNTs interlayer and a PDMS/CNTs/CBs functional layer), which effectively coordinates changes in the conductive pathway during stretching, resulting in an excellent linearity of up to 0.992 within a strain range of 100%. More importantly, owing to the quasi-homogeneous material system that minimizes mechanical mismatch, the sensor exhibits outstanding cyclic stability. After 30,000 cycles at 25% tensile strain, the sensing performance remained stable, and no significant damage was observed in the surface morphology (Figure 6d). This superior fatigue resistance, combined with its superhydrophobicity, ensures the reliability of the sensor for long-term use in complex environments.

#### 2.2.5. Mechanical Properties

**Figure 7:**
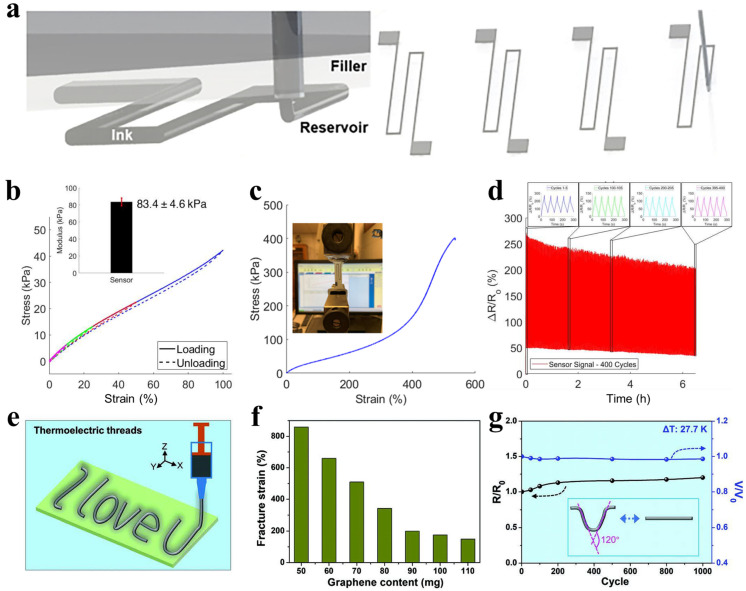
(**a**) Images and schematic diagrams of the embedded 3D printing (e−3DP) process. (**b**) Stress–strain curves and average Young’s modulus of the skin-attachable sensor. (**c**) Stress–strain curve and photograph of the sensor in the unstretched state. (**d**) Durability sensing test over 400 cycles at 50% strain. (Reproduced from [66] with permission from ScienceDirect). (**e**) Schematic illustration of the preparation of thermoelectric (TE) threads. (**f**) Fracture strain of TE threads with increasing graphene content. (**g**) Relative changes in resistance and TE voltage of the TE threads under 120° bending over 1000 cycles. (Reproduced from [67] with permission from Wiley).

Relevant mechanical properties include elastic modulus, elongation at break, breaking strength, flexibility, and fatigue life. These properties directly influence a sensor’s ability to conformally adhere to human skin, wearing comfort, and service life in dynamic environments [63,64]. The key to improving mechanical compatibility lies in employing flexible substrates and conductive materials with low modulus and high tensile flexibility [65]. Herren et al. [66] fabricated an ultra-stretchable strain sensor based on a carbon nanotube/silicone rubber nanocomposite using embedded 3D printing technology (Figure 7a). The sensor exhibits an elastic modulus as low as 83.4 kPa, which is comparable to that of human skin (∼50−150 kPa), ensuring comfortable and reliable skin adhesion. It also achieves an elongation at break of up to 535%, demonstrating ultra-stretchability. After 400 stretching cycles at 50% strain, the sensor showed excellent durability and stable resistance response, indicating remarkable fatigue resistance (Figure 7b–d). These characteristics enable reliable monitoring of large-range human joint motions, offering an effective strategy for developing high-performance skin-wearable sensors. In another study, Li et al. [67] prepared graphene/elastomer composite thermoelectric fibers via a simple 3D extrusion process (Figure 7e). The resulting fibers exhibit an elongation at break exceeding 800%, indicating ultra-stretchability. After 1000 bending cycles, the thermoelectric voltage retention remained close to 100%, while resistance increased by only about 30%, demonstrating outstanding bending fatigue resistance and electrical stability (Figure 7f,g). The fibers are soft, can be knotted without damage, and show excellent flexibility. Collectively, these mechanical properties establish the fibers as reliable candidates for wearable devices, suitable for monitoring human physiological signals and oral activities, thereby providing a material basis for self-powered sensors in long-term health monitoring.

In summary, a thorough understanding of the fundamental working mechanisms and performance parameters of strain sensors provides the essential foundation for the subsequent design and optimization of self-powered sensing systems. Different sensing mechanisms present distinct advantages and limitations across application scenarios. Thus, rational selection and structural innovation remain paramount to achieving high-performance, practical wearable sensing.

## 3. Self-Powered Strain Sensing System with Integrated Energy Storage Device

The integration of high-performance flexible energy storage devices with strain sensors represents a natural evolution from traditional electronic system design. This approach supplies stable electrical energy to sensing units through independent “miniature energy depots.” The primary advantage of this strategy is its provision of continuous and controllable power output, making it particularly suitable for applications requiring long-term continuous monitoring. Among various flexible energy storage devices, supercapacitors and micro-batteries have attracted significant research interest due to their respective performance advantages.

### 3.1. Integrated Supercapacitor

Supercapacitors store energy either through the electric double-layer effect at the electrode–electrolyte interface or via rapid surface redox reactions. They offer high power density, fast charge/discharge rates, and long cycle life, which makes them particularly well-suited for providing pulsed power to strain sensors [68,69].

**Figure 8:**
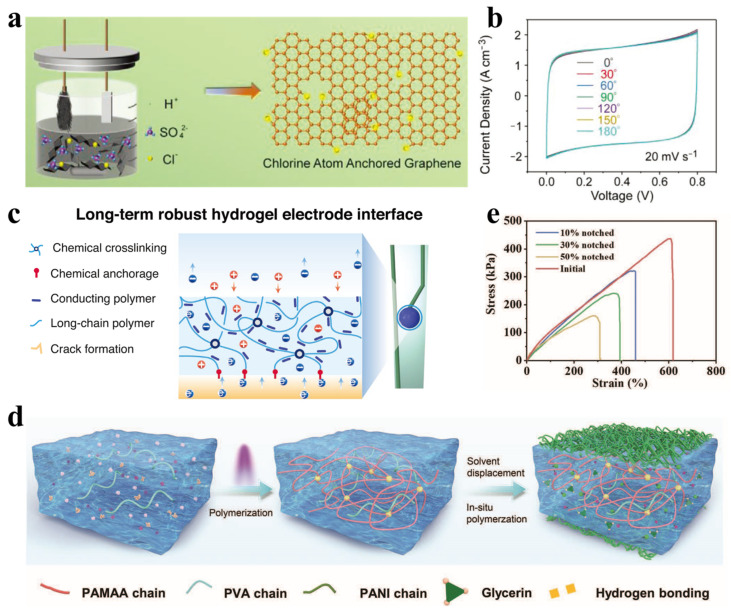
(**a**) Preparation process of Cl-G nanosheets. (**b**) CV curves of the Cl_2_-G-MSC at different bending angles. (Reproduced from [73] with permission from Wiley). (**c**) Schematic of the PEDOT:Poly(SS-4VP) hydrogel layer formed via chemical anchoring and crosslinking. (Reproduced from [79] with permission from Wiley). (**d**) Fabrication process of the all-flexible supercapacitor. (**e**) Stress–strain curves of supercapacitors with different pre-cut lengths under monotonic loading. (Reproduced from [82] with permission from Wiley).

Electrode material selection is a decisive factor for supercapacitor performance. Carbon-based nanomaterials such as graphene and carbon nanotubes, renowned for their high specific surface area, excellent electrical conductivity, and mechanical flexibility, are prevalent choices for flexible supercapacitor electrodes [70,71,72]. For example, an all-solid-state micro-supercapacitor constructed from chlorine-doped graphene—prepared via a convenient and eco-friendly one-step electrochemical exfoliation process—achieves a combination of high conductivity (13.3 S cm^−1^) with outstanding mechanical flexibility. It exhibits a capacitance retention rate of 98.9% under 180° bending, underscoring its potential for flexible electronics (Figure 8a,b) [73]. Conductive polymers such as PEDOT:PSS [74] and polyaniline [75], along with transition metal oxides like MnO_2_ [76] and RuO_2_ [77], have also been extensively investigated for their ability to deliver higher specific capacitance. Notably, composite electrodes fabricated on suitable flexible substrates can enhance mechanical flexibility while maintaining high capacitance [78]. Zhang et al. [79] developed a conductive hydrogel coating based on PEDOT:Poly(SS-4VP) using chemical anchoring and cross-linking strategies. This coating demonstrates exceptional interfacial toughness under physiological conditions, thereby effectively suppressing crack formation and delamination during repeated charge-discharge cycling (Figure 8c). The material exhibits high charge storage capacity and low interfacial impedance, while its significantly reduced Young’s modulus closely matches that of biological tissues. These combined properties facilitate a synergistic enhancement of both capacitive performance and mechanical flexibility.

Electrolyte selection is equally vital for device safety and environmental adaptability. Hydrogel electrolytes are increasingly considered an ideal option for wearable integrated systems, owing to their high ionic conductivity, excellent flexibility, and soft, wet characteristics similar to biological tissues [80]. Particularly, double-network hydrogels based on polyvinyl alcohol and polyacrylamide offer good mechanical strength and self-healing properties while maintaining high ionic conductivity, which substantially improves the reliability and service life of integrated devices [81]. Huang et al. [82] fabricated a fully flexible supercapacitor with high ionic conductivity and mechanical stability by in situ polymerization of polyaniline (PANI) on a PVA/PAM-AA double-network hydrogel (Figure 8d). The device retained structural integrity under tensile strain up to 608% and exhibited excellent anti-fatigue and anti-fracture properties (Figure 8e). Moreover, the introduction of a glycerin/water binary solvent system effectively prevented electrolyte freezing or evaporation under extreme temperatures (–60 to 100 °C), thereby ensuring stable electrochemical performance across a wide temperature range. This study provides a reliable energy solution for integrated flexible electronic systems operating under harsh environmental conditions.

**Figure 9:**
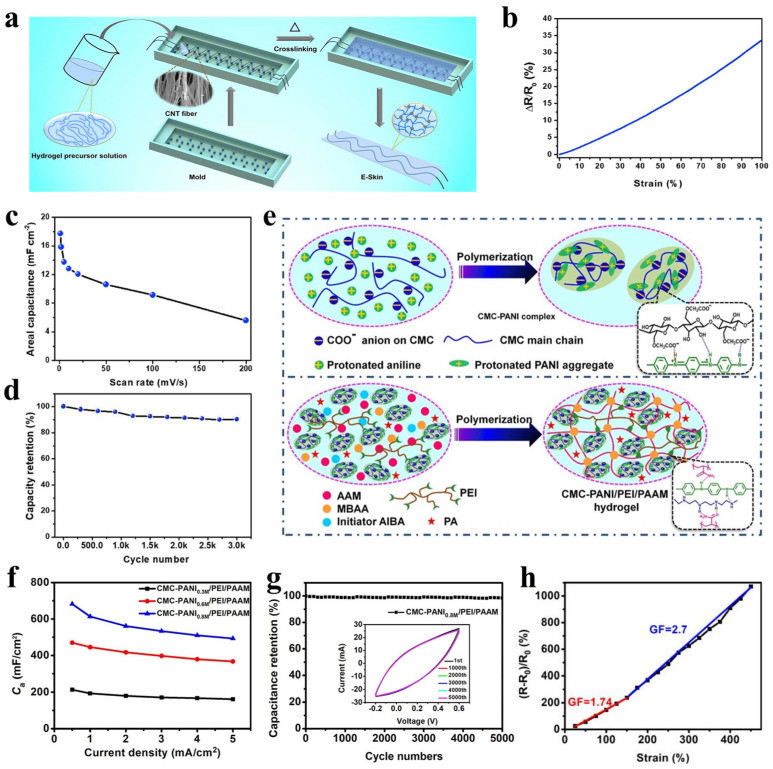
(**a**) Schematic illustration of the CFPH composite preparation. (**b**) Resistance response of the CFPH composite under tensile loading. (**c**) Areal capacitance at different scan rates. (**d**) Capacitance retention of the CFPH composite over 3000 charge-discharge cycles. (Reproduced from [86] with permission from ScienceDirect). (**e**) Preparation process of CMC-PANI/PEI/PAAM hydrogels. (**f**) Specific capacitance versus current density. (**g**) Capacitance retention of the CMC-PANI/PEI/PAAM supercapacitor after 5000 CV cycles. (**h**) Δ*R*/*R*_0_ as a function of applied tensile strain. (Reproduced from [87] with permission from ScienceDirect).

In terms of integration configuration, researchers have developed various effective structural designs. The sandwich architecture achieves high integration density by vertically stacking sensing and energy storage layers, although stress concentration between layers may cause interfacial delamination [83]. Alternatively, the planar interdigitated structure—fabricated using lithography, screen printing, or direct-write printing—places sensing and energy storage units adjacently on a unified substrate, effectively mitigating interfacial stress while potentially facing signal crosstalk challenges [84]. For electronic textiles, fiber- or yarn-based supercapacitors and strain sensors can be integrated into fabrics through weaving, knitting, or embroidery, combining wearing comfort with functionality [85]. Yi et al. [86] developed a highly stretchable carbon nanotube (CNT) fiber/PAM hydrogel composite (CFPH) that functions simultaneously as a strain sensor and a supercapacitor (Figure 9a). In this composite, CNT fibers arranged in a quasi-sinusoidal pattern serve as electrodes, while the ionically conductive PAM hydrogel acts as both electrolyte and separator, enabling strain sensing up to 100% stretch (Figure 9b). As a supercapacitor, the structure delivers a specific capacitance of 10.6 mF/cm^2^ and retains 90% capacitance after 3000 dynamic stretching cycles (Figure 9c,d). This work offers a viable route toward developing highly stretchable, transparent, and skin-adhesive multifunctional integrated systems, thereby contributing to the advancement of self-powered flexible sensors. In another study, Li et al. [87] constructed a dual-network conductive hydrogel based on polyethyleneimine/polyacrylamide (CMC-PANI/PEI/PAM) through in-situ PANI polymerization assisted by carboxymethyl cellulose (CMC) (Figure 9e). The hydrogel exhibits outstanding mechanical stretchability (>600% strain) and high electrical conductivity (61 S m^−1^). As a supercapacitor electrode, it achieves an areal capacitance of 679 mF cm^−2^ and maintains 98% capacitance retention over 5000 cycles (Figure 9f,g). Owing to its notable piezoresistive effect, the hydrogel also functions as a strain sensor with a high gauge factor (*GF* = 1.74–2.7) within a linear range of 0–450% strain (Figure 9h). The integration of the supercapacitor with the sensor resulted in a self-powered sensing system that monitors various human motions in real time without an external power supply. This study provides a feasible strategy for developing multifunctional self-powered flexible electronics operable over a wide temperature range.

### 3.2. Integrated Micro Battery

Although supercapacitors exhibit high power density, their energy density is typically lower than that of batteries, posing a challenge for meeting the energy requirements for long-term continuous operation. In contrast, flexible micro-batteries offer high energy density, rendering them ideal for applications with high energy demands.

#### 3.2.1. Material Systems and Flexibility Strategies

**Figure 10:**
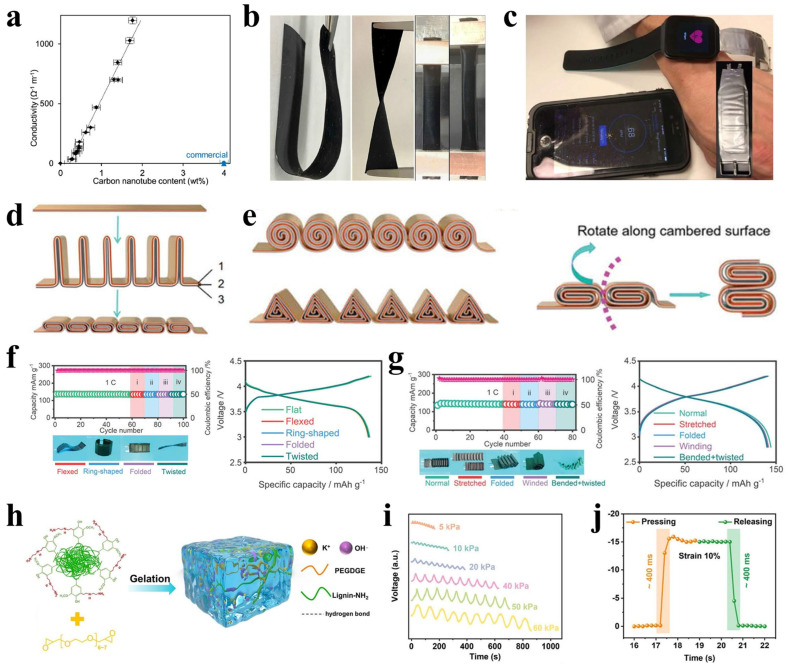
(**a**–**c**) Self-supporting SWNT-based flexible Li-ion battery: (**a**) The conductivity of self-supporting electrodes. (**b**) Demonstration of mechanical flexibility under bending, twisting, and stretching. (**c**) A wrist-worn flexible battery powering a smartwatch with real-time heart rate monitoring. (Reproduced from [95] with permission from ScienceDirect). (**d**–**g**) Bioinspired flexible battery design mimicking human joint anatomy: (**d**) Schematic of the fabrication process for flexible battery units. (**e**) Structural designs featuring cylindrical, triangular prism, and cubic configurations. (**f**) Charge/discharge cycling tests of cubic-unit cells under different configurations and corresponding galvanostatic charge–discharge profiles. (**g**) Charge/discharge cycling tests of cylindrical-unit cells under different configurations and corresponding galvanostatic charge–discharge profiles. (Reproduced from [100] with permission from Royal Society of Chemistry). (**h**–**j**) Lignin-based ionic hydrogel for self-powered Zn/Ag battery-type sensor: (**h**) Design strategy of L-IHs. (**i**) Voltage variations of the self-powered sensor under different mechanical pressures. (**j**) Resistance response of the 2.5L-2IH strain sensor during loading and unloading at 10% strain. (Reproduced from [102] with permission from ScienceDirect).

To enable integration with strain sensors, flexible micro-batteries must achieve high energy density while maintaining mechanical compliance and deformation tolerance. Promising systems such as zinc–silver, lithium-ion, and emerging zinc–air batteries are being actively developed in flexible forms [88,89,90]. Strategies for achieving battery flexibility primarily involve the use of flexible current collectors, the development of solid-state electrolytes, and innovative structural designs. Materials such as carbon cloth, graphene films, and carbon nanotube paper can replace traditional metal current collectors due to their high electrical conductivity and mechanical flexibility [91,92]. Solid polymer electrolytes, including polyethylene oxide (PEO) [93] and PAN-based systems [94], not only eliminate leakage risks but also offer excellent interfacial compatibility. In terms of flexible electrode materials, Kuznetsov et al. [95] realized high energy density and structural integration in flexible lithium-ion batteries by fabricating self-supporting electrodes based on single-walled carbon nanotubes (SWNTs). The authors introduced a solvent-free preparation process in which SWNTs are directly mixed with active materials via gas-phase mixing, thus circumventing the need for conventional metal current collectors, binders, and conductive additives (Figure 10a). The resulting electrodes retained excellent electrochemical performance under stretching, bending, and twisting, and leveraged the piezoresistive effect of the SWNT network to enable in situ structural health monitoring (Figure 10b,c). This strategy provides new materials and technologies for developing high-energy-density flexible batteries for wearable devices.

In structural design, several bio-inspired or geometry-based configurations have been proposed. The wavy structure absorbs mechanical stress through geometric deformation rather than material strain when stretched [96]. The island-bridge architecture interconnects rigid functional units with stretchable links, enabling system-level stretchability [97]. Kirigami- and origami-inspired designs employ out-of-plane configuration changes to accommodate complex deformations [98,99]. Inspired by human joint anatomy, Chen et al. [100] proposed a novel structural design for flexible lithium-ion batteries that combines thick, rigid energy storage units with thin, flexible connectors, mimicking the joint surface–ligament structure (Figure 10d,e). Finite element simulations confirmed that the structure effectively disperses stress and maintains the metal current collector within the elastic deformation range, avoiding plastic failure. The cubic-configuration battery achieves a volumetric energy density of up to 371.9 Wh L^−1^, equivalent to 92.9% of conventional aluminum-plastic packaged batteries, while the cylindrical configuration exhibits superior stretching and multi-mode deformation capability (Figure 10f,g). This structural strategy significantly enhances the mechanical durability and deformation adaptability of flexible batteries without compromising energy density, offering a reliable energy solution for wearable electronics.

#### 3.2.2. Integration Challenges and Technological Breakthroughs

The integration of batteries with sensors presents several key challenges. First, battery packaging must prevent electrolyte leakage and active material detachment under repeated mechanical deformation [101]. Second, thermal management during charge–discharge cycles is necessary to avoid affecting sensitive sensing components. Furthermore, interfacial stability between different functional materials remains critical to the system’s cycling life. Recently, Mu et al. [102] reported a technological advance in flexible self-powered sensors by fabricating a lignin-based ionic hydrogel (L-IHs). This material was synthesized via one-step free radical polymerization using amine-activated lignin (lignin-NH_2_) as a dynamic crosslinking agent and polyethylene glycol diglycidyl ether (PEGDGE) in an alkaline environment (Figure 10h). The resulting three-dimensional crosslinked network endowed the hydrogel with excellent compressibility (up to 294.56 kPa) and high ionic conductivity (2.501 mS cm^−1^). Based on this material, the researchers constructed a Zn/Ag primary battery-type self-powered sensor using L-IHs as the electrolyte. The device delivered a stable voltage output (approximately –1.48 V), a broad pressure-sensing range (5–60 kPa), and a fast response time (~400 ms) (Figure 10i,j). This system demonstrates promising potential in human–computer interaction and motion monitoring, while also opening a new pathway for the high-value utilization of biomass resources.

The primary advantage of this integrated strategy lies in its mature technical foundation and stable energy output characteristics, which facilitate standardized system design and performance optimization. However, core challenges remain in further improving the energy density and cycling stability of energy storage units under large deformation, while ensuring overall system safety and durability under complex mechanical conditions. Future research may focus on developing new flexible battery systems with higher energy density, optimizing interfacial stress management, and enhancing overall energy efficiency.

## 4. Self-Powered Strain Sensing System Based on Nanogenerators

Nanogenerators directly convert mechanical energy from human motion into electricity, providing an immediate power source for sensors and thereby eliminating dependency on external power sources. Based on their working mechanisms, they are primarily categorized as triboelectric nanogenerators (TENGs) or piezoelectric nanogenerators (PENGs) [103]. Regarding system integration, nanogenerator-based self-powered strategies can be classified into two main approaches: discrete integration and monolithic integration. In discrete integration, the nanogenerator functions as an independent energy harvester; its generated electrical power is conditioned to drive a separate sensing unit. In monolithic integration, the nanogenerator itself acts as the sensing element, with its electrical output (voltage, current, or charge) directly varying in response to mechanical stimuli, thereby achieving self-powered sensing without requiring a discrete sensor. This distinction provides a useful framework for understanding the design and functionality of nanogenerator-integrated systems discussed in the following subsections.

### 4.1. Integrated Triboelectric Nanogenerator

**Figure 11:**
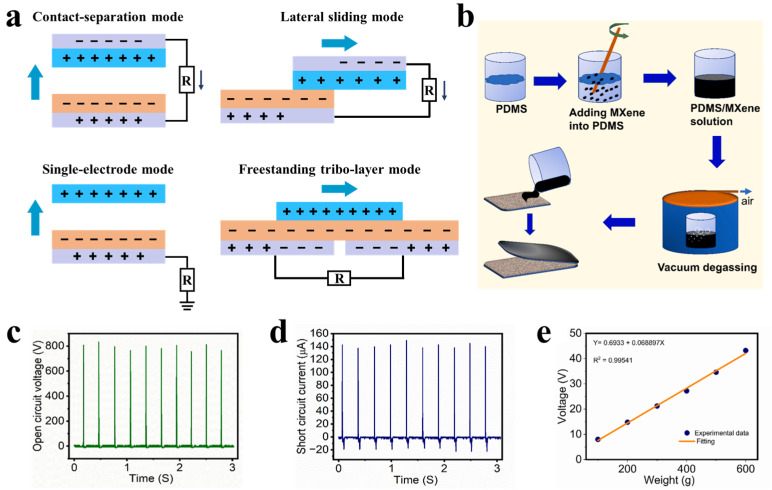
(**a**) Four fundamental modes of TENGs. (**b**) The preparation of wrinkled PDMS/MXene film. (**c**) Open circuit voltage and (**d**) short circuit current of 6W device. (**e**) Linear relationship of output voltage with different weights. (Reproduced from [111] with permission from Elsevier).

Triboelectric nanogenerators (TENGs) operate through the coupled effects of contact electrification and electrostatic induction, efficiently converting mechanical energy into alternating current signals. They are particularly suitable for harvesting low-frequency and irregular mechanical energy from human movement [104,105,106]. TENGs operate in four fundamental modes—vertical contact-separation, lateral sliding, single-electrode, and freestanding triboelectric-layer—each offering flexibility for integration into different application scenarios (Figure 11a) [107,108]. The vertical contact-separation mode is suitable for collecting energy from pressing or impacts; the lateral sliding mode applies to in-plane motions such as rotation or sliding; the single-electrode mode simplifies system structure and is ideal for harvesting energy from human motion; and the freestanding mode is effective for energy harvesting from moving objects.

As a self-powered sensor, the triboelectric nanogenerator operates not only as an energy harvester but also as a direct mechanical stimulus transducer. The sensing mechanism is based on the coupling of contact electrification and electrostatic induction. When an external mechanical stimulus (e.g., stretching, pressing, or sliding) is applied, the relative displacement or contact-separation between two triboelectric layers with opposite charge polarities induces a change in the electric potential distribution, thereby generating a measurable electrical signal (voltage or current) in the external circuit [109]. The output signal depends strongly on the strain amplitude, frequency, and deformation mode. For instance, in the vertical contact-separation mode, the output voltage is proportional to the separation distance, while in the lateral sliding mode, the signal correlates with the sliding displacement or speed. This inherent relationship between mechanical input and electrical output enables TENGs to function as self-powered strain sensors without requiring an external power supply. Moreover, the structural design of TENGs can be tailored to enhance sensitivity, linearity, and dynamic response [110]. By calibrating the electrical output against applied strain, TENG-based sensors can quantitatively monitor human motions, physiological signals, and environmental mechanical changes in real time, making them highly suitable for wearable and implantable applications.

**Figure 12:**
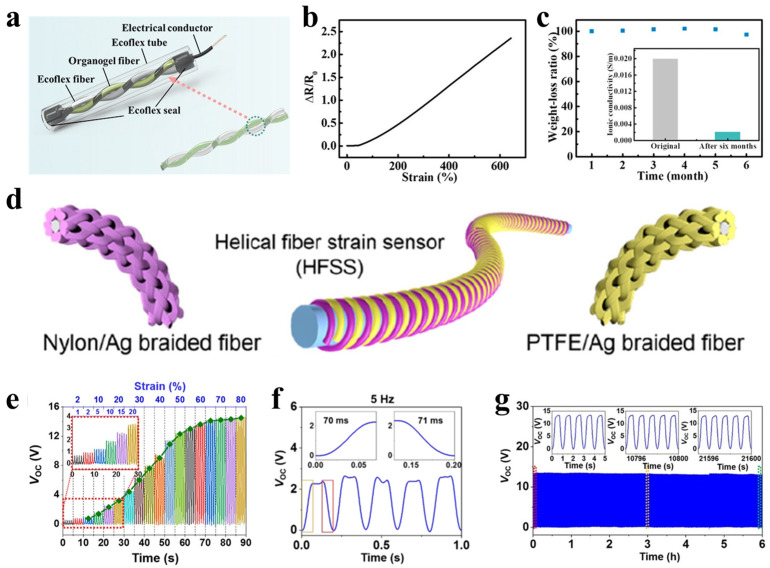
(**a**) Architecture of the OFS-TENG. (**b**) Real-time resistance variation of the organogel under different tensile strains. (**c**) Solvent retention property of the organogel after six months. (Reproduced from [112] with permission from ACS Publications). (**d**) Schematic structure of the helical-structured HFSS. (**e**) Voc of the HFSSs at tensile strains ranging from 1% to 80%. (**f**) Response time of the HFSS at a stretching frequency of 5 Hz. (**g**) Output stability and reliability of the HFSS. (Reproduced from [14] with permission from ACS Publications).

Beyond elaborate structural designs, simple and cost-effective TENG configurations have also shown substantial promise for self-powered strain sensing. For instance, Aiswarya et al. [111] fabricated a wrinkled PDMS/MXene composite-based TENG using a straightforward sandpaper templating method (Figure 11b). The engineered surface microstructure markedly increased the effective contact area and triboelectric output, yielding an open-circuit voltage of ~790 V and a short-circuit current of 140 µA (Figure 11c,d). Furthermore, the device operated as a self-powered weighing sensor, demonstrating a linear voltage response to applied loads ranging from 100 to 600 g (Figure 11e). This study illustrates that simple material and structural modifications can yield highly efficient TENGs for self-powered sensing, providing a practical and scalable pathway for wearable electronics. Sheng et al. [112] developed an implantable self-powered sensor (OFS-TENG) based on a double-helix structure comprising organic gel fibers and silicone fibers. During stretching and compression, contact-separation between the helical fibers generates electrical signals via triboelectrification and electrostatic induction, achieving mechanical-to-electrical conversion without an external power source. The device features a fiber spiral winding structure encapsulated in a biocompatible silicone tube, achieving high stretchability (600%), long-term stability (>6 months), and excellent biocompatibility (Figure 12a–c). When implanted in a rabbit’s patellar ligament, the sensor successfully facilitated in vivo real-time strain monitoring, demonstrating its potential for implantable self-powered sensing and rehabilitation training.

In line with the integration approaches described in Section 4, TENG-based systems can be implemented either as discrete power sources or as monolithic sensing units. In discrete configurations, the TENG acts as an independent energy harvester, with its rectified output supplying power to a separate sensor [113]. In monolithic configurations, the TENG itself serves as the sensor, where its electrical output (voltage, current, or charge) is directly modulated by the applied mechanical stimulus, thereby enabling self-powered strain sensing [114]. Ning et al. [14] developed a helical fiber-structured triboelectric nanogenerator (HFSS) (Figure 12d), in which the output voltage correlates directly with external tensile strain (1–80%) and frequency (0.25–4 Hz), enabling self-powered strain sensing without an external power supply. The device generates electrical signals through periodic contact-separation between PTFE and nylon triboelectric layers within the helical fiber structure, exhibiting high sensitivity (0.5 V output at 1% strain), fast response (~70 ms), and excellent mechanical stability (>20,000 cycles) (Figure 12e–g). Integrated into a chest strap, the HFSS enabled real-time monitoring of breathing patterns, heart rate, and joint bending angles, and supported the construction of a respiratory monitoring system with an intelligent alarm function. This work highlights the advantage of TENGs in directly converting mechanical stimuli into quantifiable electrical signals, advancing the development of self-powered wearable sensing technology.

### 4.2. Integrated Piezoelectric Nanogenerator

The piezoelectric nanogenerator (PENG) operates based on the direct piezoelectric effect, whereby mechanical deformation of a piezoelectric crystal induces asymmetric lattice displacement, leading to polarization and the generation of an electric potential across the material, thereby converting mechanical energy into electrical energy [115,116].

**Figure 13:**
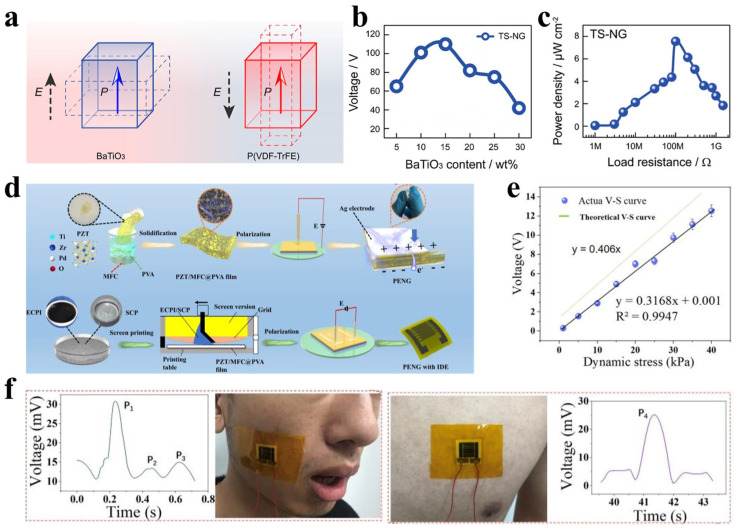
(**a**) Schematic illustrating the correlation between piezoelectric direction (P) and applied electric field (E) in BaTiO_3_ and P(VDF−TrFE) within the two-step polarized composite nanogenerator (TS-NG). (**b**) Output voltage of TS-NG films with varying BaTiO_3_ content. (**c**) Power density of TS-NG under different load resistances. (Reproduced from [123] with permission from ACS Publications). (**d**) Preparation process of PZT/MFC@PVA film and PENG sensors. (**e**) Sensitivity test of the PENG. (**f**) Output curves for facial activity and chest breathing monitoring. (Reproduced from [125] with permission from ScienceDirect).

Common piezoelectric materials are primarily classified into three categories: inorganic piezoelectric ceramics (e.g., lead zirconate titanate (PZT) [117], BaTiO_3_ [118]), piezoelectric polymers (e.g., PVDF and its copolymers [119,120]), and piezoelectric semiconductors (e.g., ZnO nanomaterials [121]). While ceramic materials such as PZT exhibit high piezoelectric coefficients, their inherent brittleness limits their use in flexible devices. In contrast, PVDF and its copolymers offer lower piezoelectric coefficients but superior flexibility, biocompatibility, and processability. ZnO nanomaterials are widely employed in micro-nano energy systems due to their unique semiconductor properties and straightforward synthesis [122]. Organic/inorganic composite materials have garnered interest for PENGs as they combine high electrical performance with mechanical flexibility. However, the negative piezoelectric effect in specific polymer components, such as poly(vinylidene fluoride-trifluoroethylene) (P(VDF-TrFE)), can diminish the overall output. To address this, Guo et al. [123] proposed a two-step polarization strategy to sequentially align the dipole orientations of BaTiO_3_ and P(VDF-TrFE), synchronizing their piezoelectric coefficient directions. This approach significantly enhances the output voltage and power density of the composite nanogenerator (Figure 13a–c). The method applies to other organic/inorganic hybrid systems, offering a general optimization route for high-performance piezoelectric energy harvesters.

The output signal of a PENG is inherently a transient pulse, making it particularly suitable for monitoring dynamic and high-frequency mechanical stimuli such as vascular pulsation, vocal cord vibration, and muscle contraction [124]. Guan et al. [125] developed a wearable PENG based on a composite of PZT, microfibrillated cellulose (MFC), and polyvinyl alcohol (PVA). The device optimizes energy harvesting and sensing performance through material selection and structural design (Figure 13d). PZT serves as an inorganic piezoelectric filler that provides high electrical output, while MFC and PVA form a hydrogen-bonded cross-linked matrix to enhance mechanical strength, flexibility, and dielectric properties. Operating via the direct piezoelectric effect, the composite film generates an electric potential under external stress through both polarization changes in PZT particles and stress transmission within the matrix. This nanogenerator demonstrates excellent performance in physiological monitoring, enabling real-time detection of weak signals such as facial muscle activity and chest breathing with a sensitivity of 0.3168 V·kPa^−1^ and a fast response time of 54 ms (Figure 13e,f), highlighting its potential for medical detection and wearable health monitoring.

The nanogenerator integration strategy enables devices to continuously harvest energy from their operating environment, significantly enhancing system autonomy and operational longevity. However, several technical challenges remain: unstable energy output can limit application in precision measurement; high internal impedance and mismatch with sensor components reduce energy transfer efficiency; and inadequate response to static or low-frequency mechanical signals restricts their use in certain types of physiological monitoring.

## 5. Integrated Self-Powered Energy Management System

As wearable electronics evolve toward lightweight, flexible designs and extended operational lifetimes, traditional single-mode energy supply approaches have become inadequate to meet the dual requirements of energy sustainability and stability in complex application scenarios. Although strategies integrating energy storage units or energy harvesters have partially alleviated power supply challenges, issues such as unstable output, limited storage capacity, and excessive system size persist. In this context, integrated self-powered energy management systems have emerged. The core concept involves co-integrating energy harvesting, storage, and management modules on a single flexible platform to form an efficient, stable, and intelligent micro-energy system. This achieves a closed-loop “harvest–store–use” cycle for environmental energy, representing the highest level of self-powered strain sensing technology.

### 5.1. System Architecture and Energy Management Strategy

A typical integrated self-powered system comprises three functional modules—the energy harvesting module, the energy storage module, and the sensing and signal processing module—coordinated through an energy management circuit. The energy harvesting module acts as the system’s “energy input port”, capturing ambient mechanical, thermal, or optical energy [126]. Common mechanical harvesters include TENGs and PENGs, which utilize contact electrification and piezoelectric effects, respectively, to convert mechanical energy from human motion or muscle vibration into electricity [127,128]. Flexible thermoelectric generators and perovskite solar cells can also be incorporated to harness temperature gradients and light radiation, forming a multi-source complementary power network [129]. The energy storage module functions as the system’s “energy buffer”, storing intermittent electricity generated by harvesters and releasing it steadily when required. Flexible supercapacitors and micro-batteries are commonly used—the former offering high power density and long cycle life, and the latter providing higher energy density suitable for long-term continuous monitoring, with selection optimized based on power demands and spatial constraints [130,131,132]. The energy management circuit, which includes rectifiers, power management ICs, and energy storage management chips, is essential for efficient energy utilization. Current research focuses on developing flexible, stretchable power management ICs to replace rigid components, thereby improving overall flexibility and wearability [133]. The sensing module serves as the “functional execution unit”, converting mechanical deformation into measurable electrical signals, with its performance directly determining the monitoring capability of the entire system [35].

### 5.2. Innovative Integrated Designs and Technological Breakthroughs

Recent research has achieved notable progress in system integration, structural simplification, and energy management optimization.

#### 5.2.1. Shared Electrodes and Material Design

**Figure 14:**
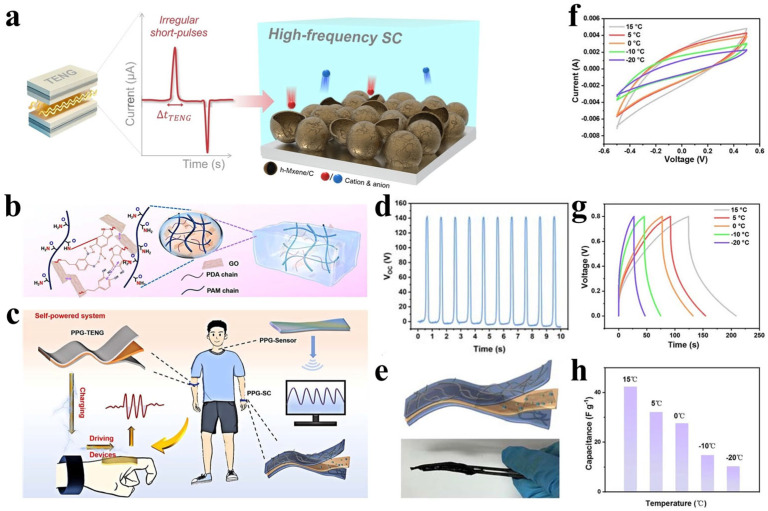
(**a**) Schematic illustration of the electrochemical interaction between TENG and high-frequency supercapacitors. (Reproduced from [135] with permission from Springer Nature). (**b**) Diagram of the internal chemical structure of the hydrogel, and (**c**) multifunctional applications of the PPG hydrogel in TENGs, supercapacitors, and strain sensors. (**d**) Voc of the TENG. (**e**) Structure and optical image of the supercapacitor. (**f**) CV curves, (**g**) galvanostatic charge–discharge profiles, and (**h**) corresponding capacitance of the PPGK hydrogel-based supercapacitor at different temperatures. (Reproduced from [138] with permission from Royal Society of Chemistry).

Allowing energy harvesters and storage units to share specific electrodes or active materials can significantly simplify fabrication, reduce interfacial resistance, and improve compactness [134]. For example, Lee et al. [135] realized integrated energy harvesting and storage by designing a shared electrode structure based on three-dimensional hollow MXene/carbon composites, which functioned simultaneously as the triboelectric layer of a TENG and the electrode material of a supercapacitor (Figure 14a). This strategy simplified device fabrication, minimized interface resistance, and enhanced both compactness and energy conversion efficiency—resulting in a charging efficiency approximately double that of conventional supercapacitors. This work provides essential material and design insights for highly integrated and efficient self-powered systems.

#### 5.2.2. Multifunctional Integrated Devices

A more innovative approach involves developing a single device capable of simultaneously harvesting, storing energy, and sensing [136,137]. Wang et al. [138] integrated these three functions into one device using a multifunctional dual-network conductive hydrogel (PPG) based on polydopamine/polyacrylamide/graphene oxide (PDA/PAM/GO). The hydrogel exhibits high conductivity (0.173 S cm^−1^), ultra-stretchability (~1100%), and self-adhesiveness. It can serve as the TENG electrode, supercapacitor electrolyte, and piezoresistive strain-sensing layer (Figure 14b,c). The resulting PPG-TENG outputs an open-circuit voltage up to 143 V in contact–separation mode while detecting various human motions and physiological signals (Figure 14d). Meanwhile, the supercapacitor assembled with this hydrogel electrolyte maintains good capacitive performance across a wide temperature range, including –20 °C (Figure 14e–h). By integrating the TENG with the supercapacitor, a self-charging power system was constructed, achieving closed-loop mechanical energy harvesting, storage, and sensing. This provides a feasible material and device platform for all-weather self-powered wearable health monitoring.

Further extending the concept of multifunctionality, recent research has demonstrated the integration of energy harvesting with active environmental remediation capabilities. Pandiyan et al. developed reinforced spontaneously polarized nanofibers (RSNF) by incorporating size-confined and surface-tailored MXene nanosheets into a poly(vinylidene fluoride-co-hexafluoropropylene) (PVDF-HFP) matrix via electrospinning [139]. The RSNF membrane serves not only as a high-performance piezoelectric nanogenerator, delivering an output voltage of ~27.8 V under biomechanical stimulation, but also exhibits remarkable photo-piezocatalytic activity. Under combined mechanical vibration and solar irradiation, the membrane achieved 90% degradation of Rhodamine B dye within 120 min, showcasing a synergistic piezo-photocatalytic effect that surpasses individual catalytic processes. This work exemplifies an advanced integration strategy where the energy harvesting unit inherently possesses environmental interaction functionality, broadening the application scope of self-powered systems beyond sensing to include sustainable remediation technologies.

#### 5.2.3. Intelligent Energy Management Technology

To further improve energy utilization efficiency, researchers have introduced intelligent energy management strategies based on activity recognition. These systems employ microcontrollers to monitor the user’s movement status and ambient energy availability in real time, dynamically adjusting energy allocation. For instance, when high activity levels are detected, the system prioritizes power delivery to sensors to ensure critical data acquisition; during low activity, it shifts focus to energy storage, reserving power for subsequent monitoring tasks. This activity-aware energy management approach enhances overall system efficiency and significantly extends autonomous operation time. Zi et al. [140] proposed an optimized charging strategy using motion-triggered switching, substantially improving TENG charging efficiency for energy storage units. By introducing a “designed charging cycle,” the system actively triggers a short circuit when the TENG reaches its displacement limit, facilitating complete charge transfer and increasing the amount of charge delivered to the storage unit per cycle. This dynamic energy management method raises maximum energy storage efficiency from 25% with conventional rectifier charging to 50%, and at least doubles the saturation voltage, enabling efficient regulation and allocation of harvested energy. This work provides important insights for dynamically optimizing energy flow in self-powered systems based on mechanical motion states.

Although the integrated “harvest–store–use” system represents the highest level of self-powered technology and enables truly long-term autonomous operation, several technical challenges remain. The development of flexible energy management circuits lags behind other functional modules; interfaces between modules require optimization for efficient connectivity and mechanical stress matching; and the robustness and long-term durability of the entire system under complex deformations need further improvement. Future research may focus on developing novel multifunctional material systems, designing bio-inspired structures for improved mechanical adaptability, investigating dynamic impedance matching technologies to enhance energy efficiency, and creating AI-adaptive energy management algorithms. As these technical bottlenecks are gradually overcome, integrated self-powered strain sensing systems are expected to play increasingly important roles in personalized medicine, intelligent human–computer interaction, and long-term health monitoring.

In recent years, self-powered strain sensors have undergone rapid development in flexible electronics and wearable devices, exhibiting diversified performance characteristics as summarized in Table 1. In terms of sensitivity, the MP@PU fiber sensor achieves a gauge factor of up to 9.95 × 10^5^ within 110–120% strain, while porous graphene foam demonstrates a gauge factor of 1401.5 at 30–45% strain, indicating exceptionally high responsiveness. Regarding sensing range, most hydrogel-based sensors (e.g., PVAA-MXene, ZIF-8@PAm/PVP) enable broad strain monitoring from 600% to 1400%, suitable for large-deformation scenarios on the human body. In contrast, sensors such as CIGS specialize in precise detection of minor strains (0–2%). In dynamic performance, CIGS and ZIB-P sensors exhibit response/recovery times as short as tens of milliseconds, making them suitable for high-frequency monitoring. For cyclic stability, the ZIB-P sensor withstands over 100,000 cycles at 14.3 kPa, while triboelectric and MP@PU sensors also exceed 2000 cycles, demonstrating excellent durability. Mechanically, various hydrogels show high fracture elongation (>1000%) with moderate strength, meeting requirements for body-worn applications. Overall, self-powered strain sensors are advancing toward the integration of high sensitivity, wide sensing range, fast response, high stability, and favorable mechanical properties. Future efforts should focus on enhancing environmental adaptability, signal consistency, and system integration to facilitate their practical deployment.

**Table 1:** Performance comparison of self-powered strain sensors in the latest literature.

Self-Powered Strain Sensing System	Sensitivity (GF)	Sensing Range	Response/Recovery Time (ms)	Cyclic Stability	Fracture Stress (MPa)/Tensile Elongation (%)
PEDOT:PSS/CNT/WPU composite film [141]	-	0–100%	300/700	300 (at 20% strain)	-/400
CQDs/MWCNT strain sensor [142]	94.1	0–80°	-/-	5000 (at 60°)	-/-
PVAA-MXene hydrogel [143]	1.10 (0–400%) 1.76 (400–800%) 2.99 (800–1400%)	0–1400%	240/-	5000 (at 100% strain)	1.2/1700
CIGS sensor [144]	10.34	0–2%	0.03/0.02	120 (at 1.2% strain)	-/-
RGO-PY@HI [145]	5.05	0–180°	-/-	200 (at 90°)	-/-
PAM-BTO/NaCl hydrogel [146]	1.52 (0–400%) 2.12 (400–600%)	0–600%	-/-	50 (at 20% strain)	-/-
PVA/PAMAA/Gly/NaCl organohydrogel [147]	1.817 (0–200%) 3.436 (200–500%) 8.303 (500–1000%)	1–1000%	160/-	600 (at 15% strain)	0.345/1002
TEC-based sensor [148]	1.03 (≤50%)	0–50% 0–9.5 kPa	-/-	1200	0.26/167
Mechano-electrochemical harvesting (MECH) fiber [149]	-	0–100%	-/-	1000 (at 100% strain)	2/>100
PcNA/MXene sensor [150]	1.86 (0–180%) 1.1 (180–300%)	0–300%	290/342	2000 (at 50% strain)	-/930
CCNC-C_3_N_4_- PAM hydrogel [151]	5.6 (0–1.6%)	0–2800%	-/-	-	0.135/2800
Piezoionic sensor [152]	-	-	100/80	>20,000	23.42/1200
Porous graphene foam-based material [153]	109.8 (0–30%) 1401.5 (30–45%)	0–45%	-/-	-	-/≥45
SnSe2/graphene heterojunction [154]	450	-	-/-	10,000 (at 0.4–0.5% strain)	-/-
MP@PU fiber sensor [155]	1.33 × 10^2^ (30%) 3.31 × 10^2^ (30–50%) 5.83 × 10^2^ (50–80%) 6.23 × 10^3^ (80–110%) 9.95 × 10^5^ (110–120%)	0–290%	400/300	2000	-/290
ZIF-8@PAm/PVP hydrogel sensor [156]	2.34 (0–200%) 4.76 (200–600%) 6.38 (600–900%)	0–900%	140/140	500 (at 200% strain)	0.328/944.2

## 6. Challenges and Prospects

Despite significant progress in self-powered wearable strain sensing systems, numerous challenges must be overcome to achieve large-scale commercial adoption. This section systematically analyzes current technical bottlenecks and proposes potential future development directions from a multidisciplinary perspective.

### 6.1. Current Main Challenges

Energy Balance Issue. The system’s total energy consumption—covering sensing, processing, and transmission—must not exceed the energy harvested from the environment. Currently, wireless data transmission is the most energy-intensive component, often surpassing the real-time supply from harvesters. Achieving ultra-low power consumption in communication design is crucial. Research focuses on energy-efficient strategies, including low-duty-cycle protocols (e.g., Bluetooth Low Energy) and event-driven transmission activated only by significant physiological events [17,22]. Additionally, on-device lightweight data processing and feature extraction can reduce data volume before transmission [6]. Future progress may involve communication-sensing co-design and adaptive transmission scheduling, which would dynamically adjust reporting rates based on energy availability to enable sustainable self-powered operation.

Mechanical Robustness and Wearability. Integrating multiple functional layers can compromise conformal skin contact and mechanical durability. Ensuring interfacial integrity between dissimilar materials under dynamic loading remains a key challenge. To prevent delamination, advanced material engineering strategies are essential. These include using chemical bonding agents (e.g., silane coupling agents) to enhance filler-polymer adhesion [44] and employing interfacial welding techniques such as π-π stacking or in-situ polymerization to strengthen heterogeneous layer bonding [62,83]. Incorporating dynamic crosslinking networks (e.g., hydrogen or ionic bonds) can further dissipate stress and improve fatigue resistance, extending the operational lifetime of wearable sensors in practical conditions.

Environmental Stability. Practical applications expose devices to sweat, temperature fluctuations, and UV radiation, all of which can degrade electrochemical, triboelectric, or mechanical properties, leading to sensor drift or system failure. For instance, moisture absorption reduces dielectric performance, temperature variations affect piezoelectric coefficients, and sweat ions interfere with electrode reactions. Developing self-healing encapsulation materials and multilayer barrier structures thus offers a promising approach to enhance environmental robustness.

Sustainable Manufacturing and Cost Control. Most laboratory prototypes rely on high-precision manufacturing techniques such as electron-beam lithography and vacuum evaporation, which involve complex processes, high costs, and limited scalability, hindering large-scale commercialization. While low-cost, high-throughput alternatives—such as inkjet printing, laser direct writing, and roll-to-roll processing—are promising, their widespread adoption in complex flexible electronics faces several limitations. These include relatively lower resolution and patterning accuracy compared to conventional lithography, challenges in material compatibility and multilayer registration, insufficient throughput for integrated multi-functional systems, and limited long-term reliability under mechanical deformation. Furthermore, the lack of standardized processes for the heterogeneous integration of energy harvesters, storage units, and sensors on a single flexible platform remains a major barrier. Overcoming these technical hurdles through material innovation, process optimization, and equipment development is essential to enable scalable, cost-effective manufacturing of robust self-powered wearable systems.

### 6.2. Future Development Directions

Multimodal Hybrid Energy Harvesting Systems. Relying on single energy harvesting methods proves insufficient for an all-weather power supply. Future systems will increasingly integrate mechanical, thermal, and solar energy harvesting—for example, by combining triboelectric nanogenerators (mechanical energy), flexible thermoelectric generators (body heat), and perovskite solar cells (ambient light). Intelligent power management circuits will enable coordinated management and efficient conversion of multiple energy sources, significantly enhancing energy autonomy in complex environments.

Flexible Microelectronics and Stretchable Power Management ICs (PMICs). As highlighted in Section 5, the development of flexible power management circuits lags significantly behind other functional modules (e.g., energy harvesters, storage units, and sensors), forming a critical bottleneck in realizing fully integrated, robust, and wearable self-powered systems. Future research must prioritize the advancement of stretchable and compliant PMICs that can efficiently regulate, convert, and distribute harvested energy while withstanding repeated mechanical deformation. Key efforts should focus on material innovation (e.g., organic semiconductors, liquid metals), architecture design (e.g., island-bridge, serpentine layouts), system-on-flex integration, dynamic impedance matching, and scalable manufacturing techniques such as printing-based fabrication. Bridging this gap will enhance energy utilization efficiency and accelerate the commercialization of truly autonomous wearable strain sensing platforms.

Intelligent Response and Information Processing. Integrating artificial intelligence with sensing systems represents a major future trend. Deploying lightweight neural network models at the device level enables local data processing and feature extraction, allowing the transmission of only compressed features rather than raw data, which substantially reduces wireless communication energy consumption. Further development of resistive memory arrays with integrated sensing, storage, and computing functions will lay the foundation for “intelligent sensing skins” with learning and adaptive capabilities.

Biocompatibility and Biodegradability. For implantable or short-term skin-adhesive applications, device biocompatibility and even biodegradability are critical. Constructing “green” transient electronics using natural biomaterials such as gelatin, cellulose, and silk fibroin—enabling harmless dissolution after service—not only avoids secondary extraction surgery but also aligns with sustainable development goals. Current research focuses on regulating material degradation kinetics and electrical properties to ensure stable performance during operation.

Exploration of New Sensing Mechanisms and Multifunctional Materials. Continued investigation of novel physical effects and advanced functional materials—such as developing lower-power sensors based on ionic conductors, or discovering biocompatible materials with higher piezoelectric/triboelectric coefficients—will provide innovative solutions for next-generation self-powered sensing systems. Meanwhile, machine learning-assisted discovery and optimization of high-performance functional materials are emerging as a key research focus in materials informatics.

## 7. Conclusions

Self-powered technology represents a key breakthrough in overcoming the energy bottleneck of wearable electronics and achieving long-term autonomous operation. This review systematically examines how the energy supply challenge is being progressively addressed through innovative integration of strain sensors with energy storage devices (supercapacitors and batteries), energy harvesters (nanogenerators), and advanced materials and structures. From independent “store–use” systems, to direct “harvest–use” configurations, and further to highly integrated “harvest–store–use” microsystems, research in this field demonstrates a clear evolution from single-function to multifunctional, from discrete to integrated, and from “active” to “truly passive” operation.

Although challenges remain in energy balance, mechanical robustness, environmental stability, and scalable manufacturing, the continuous emergence of new materials, processes, and circuit designs—particularly deep integration with artificial intelligence—will drive future self-powered strain sensing systems toward greater intelligence, comfort, and reliability. It is anticipated that through interdisciplinary collaboration, self-powered wearable strain sensing technology will play an increasingly vital role in personalized health monitoring, intelligent human–computer interaction, and soft robotics, ultimately becoming a seamless and reliable technological partner that enhances human health and capability in daily life.

## Data Availability

No new data were created or analyzed in this study.
